# Long‐Term Impact of EGUIDE Training on Facility‐Wide Guideline Adherence Rate in Schizophrenia and Major Depressive Disorder: A Multicenter Study

**DOI:** 10.1002/npr2.70067

**Published:** 2025-10-28

**Authors:** Naomi Hasegawa, Hiroyuki Muraoka, Yusuke Arai, Toru Horinouchi, Kazuhiro Yamamuro, Naoki Hashimoto, Shinichiro Ochi, Takashi Tsuboi, Yasushi Kawamata, Toshinori Nakamura, Yuka Yasuda, Junya Matsumoto, Satsuki Ito, Toshiaki Onitsuka, Kazutaka Ohi, Shusuke Numata, Hikaru Hori, Ken Inada, Koichiro Watanabe, Norio Yasui‐Furukori, Ryota Hashimoto

**Affiliations:** ^1^ Department of Pathology of Mental Diseases, National Institute of Mental Health National Center of Neurology and Psychiatry Tokyo Japan; ^2^ Department of Psychiatry Kitasato University, School of Medicine Sagamihara Kanagawa Japan; ^3^ Department of Community Mental Health Shinshu University School of Medicine Matsumoto Nagano Japan; ^4^ Department of Psychiatry Hokkaido University Graduate School of Medicine Sapporo Hokkaido Japan; ^5^ Center for Health Control Nara Medical University Kashihara Nara Japan; ^6^ Department of Psychiatry Nara Medical University, School of Medicine Kashihara Nara Japan; ^7^ Department of Neuropsychiatry, Molecules and Function Ehime University Graduate School of Medicine Toon Ehime Japan; ^8^ Department of Neuropsychiatry Kyorin University School of Medicine Tokyo Japan; ^9^ Department of Psychiatry Dokkyo Medical University School of Medicine Mibu Tochigi Japan; ^10^ Department of Psychiatry Shinshu University School of Medicine Matsumoto Nagano Japan; ^11^ Life Grow Brilliant Mental Clinic Medical Corporation Foster Osaka Japan; ^12^ National Hospital Organization Sakakibara National Hospital Tsu Mie Japan; ^13^ Department of Psychiatry Gifu University Graduate School of Medicine Gifu Japan; ^14^ Department of Psychiatry, Graduate School of Biomedical Science Tokushima University Tokushima Japan; ^15^ Department of Psychiatry, Faculty of Medicine Fukuoka University Fukuoka Japan

**Keywords:** evidence practice gap, guideline, major depressive disorder, quality indicators, schizophrenia

## Abstract

**Objective:**

The Effectiveness Research on the Dissemination and Education of Psychiatric Clinical Practice Guidelines (EGUIDE Project) was launched in Japan to promote guideline‐adherent treatment for schizophrenia and major depressive disorder (MDD) through educational outreach programs. Although short‐term effects on participating physicians have been reported, the long‐term and facility‐wide effects remain unclear. This study evaluated whether guideline‐compliant treatment behaviors improved across institutions over time, indicating potential diffusion or spillover effects.

**Methods:**

We conducted a prospective observational study involving 298 psychiatric facilities between 2016 and 2023. Discharge prescriptions and treatment data were collected for 19 623 patients with schizophrenia and 9805 patients with MDD. Adherence to the guidelines was assessed using 11 schizophrenia quality indicators (QI‐S) and seven MDD quality indicators (QI‐D). We performed logistic regression analyses, adjusting for age, sex, and facility type, with Bonferroni correction for multiple comparisons.

**Results:**

For schizophrenia, significant year‐on‐year improvements were observed in seven of the 11 QI‐S, including assessment of treatment‐resistant schizophrenia (TRS) diagnosis (from 42.2% to 62.5%), use of modified electroconvulsive therapy (mECT; from 6.1% to 11.8%), and nonprescription of anticholinergics (from 70.7% to 81.7%). For MDD, three of the seven QI‐D showed improvement, including assessment of severity diagnosis (from 51.2% to 77.0%) and use of mECT (from 12.8% to 26.6%). Notably, the implementation of cognitive behavioral therapy (CBT) decreased. These findings suggest long‐term behavioral changes across all facilities, extending even to nonparticipating clinicians.

**Conclusion:**

The presence of EGUIDE‐trained psychiatrists was associated with sustained improvements in guideline‐compliant treatments at the institutional level. These results imply not only individual educational benefits but also a diffusion of practice culture—that is, a spillover effect—leading to enhanced quality of psychiatric care. Continued educational efforts are essential to improving treatment practices at scale.

**Trial Registration:**

The protocol for the EGUIDE Project is registered with the University Hospital Medical Information Network Registry (UMIN000022645)

## Abstract 2

EGUIDE training produced sustained facility‐wide improvements in schizophrenia guideline adherence over seven years. Seven of eleven quality indicators significantly improved, including treatment‐resistant schizophrenia assessment and modified electroconvulsive therapy use, suggesting knowledge spillover effects from trained to nonparticipating psychiatrists within institutions.

## Graphical abstract

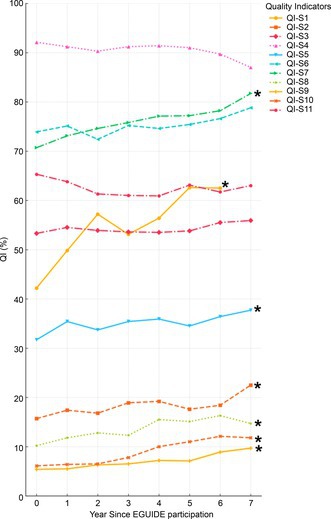

## 1 Introduction

Clinical practice guidelines are essential tools that support evidence‐based medicine (EBM) and promote shared decision‐making between patients and healthcare providers [1]. However, in real‐world clinical practice, insufficient adherence to guidelines and treatments that deviate from recommendations have been reported, highlighting the persistent gap between EBM and clinical reality [2, 3, 4]. In mental health care, guidelines for appropriate pharmacotherapy have been established. Nevertheless, the gap between EBM and clinical practice remains a challenge in psychiatric care [5]. In particular, for severe mental illnesses such as schizophrenia and depression, inappropriate polypharmacy and long‐term use of benzodiazepines have been identified as significant issues [6, 7].

To address this challenge, the Effectiveness Research on the Dissemination and Education of Psychiatric Clinical Practice Guidelines (EGUIDE Project) was initiated in Japan in 2016. This project conducts nationwide training at medical institutions, aiming to promote understanding and implementation of treatment guidelines for depression and schizophrenia in clinical practice [8, 9]. EGUIDE training, delivered through educational outreach programs by psychiatric specialists—including members of the guideline development committee—is conducted both in person and remotely. To evaluate the effectiveness of the EGUIDE training, we measured participants' clinical knowledge of the treatment guidelines and their self‐reported clinical behaviors using pre‐ and post‐training questionnaires, both of which showed improvement [10, 11, 12, 13]. Following the training, prescribing patterns and treatment implementation at each facility were evaluated, with objective feedback provided using Quality Indicators (QIs) [14]. Previous reports from the project have highlighted issues such as high rates of antipsychotic polypharmacy in schizophrenia, polypharmacy of antidepressants, inappropriate prescribing of anxiolytics or hypnotics, inappropriate co‐prescription of anticholinergics in depression, underuse of electroconvulsive therapy, and frequent as‐needed (PRN) psychotropic prescriptions [6, 7, 15, 16, 17, 18, 19, 20, 21, 22, 23, 24]. Furthermore, the project reported insufficient implementation of guideline‐recommended diagnostic processes, such as the assessment of treatment‐resistant schizophrenia and severity diagnosis in depression [25, 26]. Regarding the impact of participation in the EGUIDE training on prescribing behavior, Hasegawa et al. [14] reported that psychiatrists who participated in the training showed higher rates of antipsychotic monotherapy for schizophrenia and antidepressant monotherapy for depression than nonparticipating physicians. However, that study examined the relationship between individual physician participation and treatment behavior. The long‐term impact of EGUIDE training on the overall quality of care at participating facilities remains underexplored. We hypothesized that the presence of EGUIDE‐trained psychiatrists within a facility would promote the diffusion of their knowledge and practices to nonparticipating physicians, resulting in continuous, long‐term improvements in guideline‐compliant treatment implementation rates across the entire facility. Although the EGUIDE Project has been continuously implemented since 2016, its long‐term effects at participating facilities have not been investigated. Therefore, this study aimed to investigate the relationship between the number of years since the implementation of EGUIDE training and the implementation rates of guideline‐compliant treatments for schizophrenia and major depressive disorder, using prospective observational data.

## 2 Methods

### 2.1 Study Design

This study was a multi‐institutional, prospective study. Between 2016 and 2023, 298 medical facilities participated in the EGUIDE Project. Participation in the EGUIDE Project was voluntary for psychiatrists at participating facilities. Therefore, the proportion of participating psychiatrists varied by hospital; in total, 1421 psychiatrists participated. Written informed consent was obtained from each participating psychiatrist. Because informed consent was not obtained from nonparticipating psychiatrists, their information (number and proportion) was not collected. Discharge prescription data and inpatient treatment data for patients with schizophrenia and major depressive disorder at hospitals participating in the EGUIDE Project were collected. The primary outcome of this study was the treatment behavior of psychiatrists, as evaluated using QIs. The QIs were defined to measure adherence to guideline‐recommended treatments. This study was conducted with the approval of the National Center of Neurology and Psychiatry Ethics Committee. The protocol for the EGUIDE Project is registered with the University Hospital Medical Information Network Registry (UMIN000022645). This study was conducted in accordance with the Declaration of Helsinki of the World Medical Association.

### 2.2 Patients

Patients were diagnosed based on DSM‐5 criteria. The target patients for this study were individuals with schizophrenia or depression who were discharged from each participating facility between 2016 and 2023. At each facility, patient clinical record information was collected using an opt‐out consent process.

### 2.3 Data Collection

At each participating facility, the physician responsible for the EGUIDE facility collected data from inpatient medical charts and discharge prescriptions for all patients with schizophrenia and major depressive disorder. These physicians extracted relevant clinical information, including diagnoses, severity assessments, treatment procedures, and medication prescriptions according to the research protocol and instructions from the central administration office. Data quality was ensured through a rigorous multistep process. All collected data were submitted to the central data center at the National Center of Neurology and Psychiatry, where comprehensive quality control (QC) procedures were implemented. The QC process included manual error screening for missing values, logical consistency checks between related variables, and range validation for numerical data. When discrepancies or inconsistencies were identified, formal queries were issued to the responsible physicians for verification and correction.

### 2.4 QIs

QIs operationalize guideline‐recommended treatments at the facility level and are calculated from inpatient records using medical charts and discharge prescriptions. For example, the antipsychotic monotherapy rate in schizophrenia (QI‐S3) is calculated as the number of inpatients with schizophrenia discharged on antipsychotic monotherapy divided by all inpatients with schizophrenia during the assessment period, expressed as a percentage. Higher values for all QIs indicate better adherence to guideline recommendations. QIs were defined within the EGUIDE Project to evaluate adherence to recommended treatments outlined in psychiatric practice guidelines. Currently, 11 indicators have been established for schizophrenia and seven for depression (Table 1 and Table S2). Each QI data was collected for up to eight years, spanning from before the training to seven years after the training. However, for QI‐S1 (assessment of treatment‐resistant schizophrenia [TRS] diagnosis) and QI‐D1 (assessment of severity diagnosis), data collection began in 2017, so a maximum of seven years of data was collected.

**TABLE 1:** Demographic and clinical characteristics in patients.

	Schizophrenia	Major depressive disorder
Number of patients	*n* = 19623	*n* = 9805
Age, mean (SD) years	46.9 (15.7)	58.3 (18.6)
Sex, no. (%)		
Male	8689 (44.3)	3219 (32.8)
Female	10 934 (55.7)	6586 (67.2)
Institution, no. (%)		
University hospitals	6020 (30.6)	4990 (50.8)
Public hospitals	6863 (35.0)	2193 (22.4)
Private hospitals	6740 (34.3)	2622 (26.7)
Number of patients per elapsed year, no. (%)		
Before participating	3050 (15.5)	1441 (14.7)
After 1 year	3806 (19.4)	1823 (18.6)
After 2 years	3285 (16.7)	1725 (17.6)
After 3 years	2857 (14.6)	1419 (14.5)
After 4 years	2439 (12.4)	1152 (11.7)
After 5 years	1981 (10.1)	952 (9.7)
After 6 years	1267 (6.5)	721 (7.4)
After 7 years	938 (4.8)	572 (5.8)

Abbreviation: SD, standard deviation.

### 2.5 Statistical Analysis

All statistical analyses were performed using SPSS version 30 (IBM Corp., Armonk, NY). A significance level of *p* < 0.05 was adopted. To prevent Type I errors caused by multiple comparisons, a Bonferroni correction was applied to the analysis. As 36 analyses were performed in this study, the corrected significance level was set at *p* < 0.0014 (0.05/36). Age, sex, and facility characteristics were included as covariates in the logistic regression analysis to adjust for their known influences on the QIs [7, 15].

## 3 Results

### 3.1 Demographic and Clinical Characteristics

The participants of this study included 19 623 patients with schizophrenia and 9805 patients with major depressive disorder. The demographic and clinical characteristics of each patient group are shown in Table 1. The average age of patients with schizophrenia was 46.9 ± 15.7 years, and 44.3% were male. Among the facility types, university hospitals had the lowest proportion (30.6%). The average age of patients with major depressive disorder was 58.3 ± 18.6 years, and 32.8% were male. Among the facility types, university hospitals had the highest proportion (50.8%). Details regarding the number of patients with schizophrenia per year of participation in the EGUIDE training and the number of patients with major depressive disorder in each year following participation in the training are provided in Tables S3 and S4, respectively.

### 3.2 Annual Changes in the QIs by Years of Participation in the EGUIDE Project

#### 3.2.1 Schizophrenia

**FIGURE 1:**
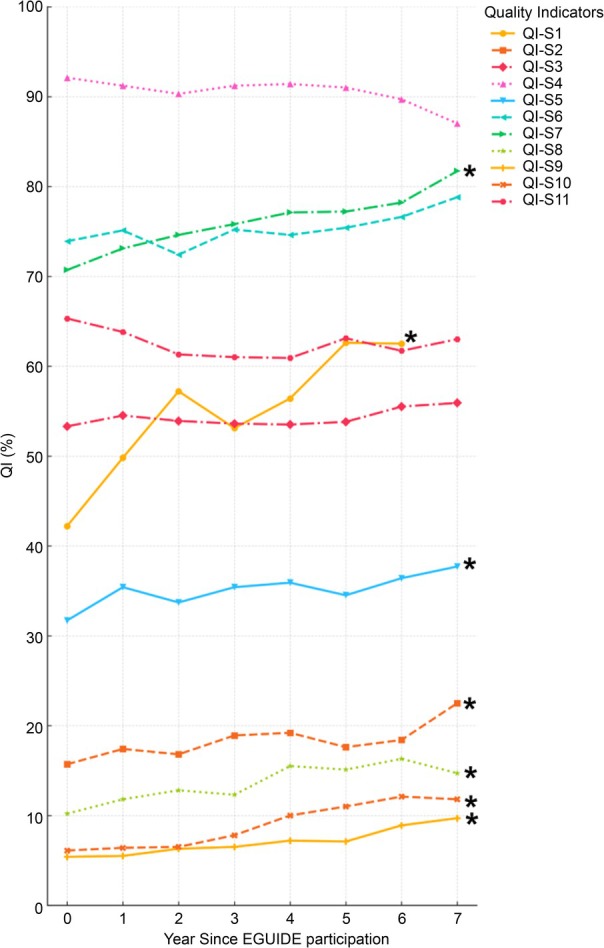
Trends in quality indicators for schizophrenia treatment over the pre‐participation period and seven years post‐participation. This figure depicts the quality indicators (QIs) for patients with schizophrenia enrolled in the EGUIDE Project. Data were collected from the period immediately preceding the intervention (Year 0) and for seven years following the intervention. The QIs included QI‐S1 (assessment of treatment‐resistant schizophrenia diagnosis), QI‐S2 (antipsychotic monotherapy without other psychotropics), QI‐S3 (antipsychotic monotherapy), QI‐S4 (no prescription of antidepressants), QI‐S5 (no prescription of anxiolytics or hypnotics), QI‐S6 (no prescription of mood stabilizers or antiepileptics), QI‐S7 (no prescription of anticholinergics), QI‐S8 (use of long‐acting injectable antipsychotics), QI‐S9 (treatment with Clozapine), QI‐S10 (modified electroconvulsive therapy), and QI‐S11 (no prescription of psychotropic PRN medications). Notably, data for QI‐S1 were collected starting in 2017, resulting in seven years of data collection for this specific indicator. The total number of patients was 19 623, and the facility types were university hospitals (30.6%), public hospitals (35.0%), and private hospitals (34.3%). The QIs marked with an asterisk (*) indicate a statistically significant change over time, as determined by logistic regression analysis adjusted for age, sex, and facility type (*p* < 0.0014; Bonferroni‐corrected threshold: 0.05/36 comparisons).

Logistic regression analysis was conducted to examine the association between participation in the EGUIDE training (as the independent variable) and each QI for patients with schizophrenia, adjusting for age, sex, and facility type. The analysis revealed significant improvements in seven QIs: QI‐S1, QI‐S2 (antipsychotic monotherapy without other psychotropics), QI‐S5 (no prescription of anxiolytics or hypnotics), QI‐S7 (no prescription of anticholinergics), QI‐S8 (use of long‐acting injectable antipsychotics), QI‐S9 (treatment with Clozapine), and QI‐S10 (modified electroconvulsive therapy [mECT]) (all *p* < 1.4 × 10^−3^) (Table 2). Specifically, for the top three items with the smallest *p* values, QI‐S1 increased from 42.2% to 62.5% (OR 1.14, 95% CI 1.12–1.16, *p* = 4.6 × 10^−55^), QI‐S10 increased from 6.1% to 11.8% (OR 1.11, 95% CI 1.08–1.14, *p* = 1.1 × 10^−16^), and QI‐S7 increased from 70.7% to 81.7% (OR 1.08, 95% CI 1.06–1.10, *p* = 5.6 × 10^−21^) (Figure 1, Table 2).

**TABLE 2:** Longitudinal changes in QIs following institutional participation in the EGUIDE project for schizophrenia.

QIs	Unadjusted OR [95% CI]	*p*	Adjusted OR [95% CI]	*p*
QI‐S1: Proportion of assessment of TRS diagnosis	1.14 [1.13–1.16]	**2.8 × 10** ^**−60**^	1.14 [1.12–1.16]	**4.6 × 10** ^**−55**^
QI‐S2: Proportion of antipsychotic monotherapy without other psychotropics	1.04 [1.02–1.06]	**1.4 × 10** ^**−5**^	1.05 [1.03–1.07]	**2.7 × 10** ^**−7**^
QI‐S3: Proportion of antipsychotic monotherapy	1.01 [0.99–1.02]	0.35	1.01 [1.00–1.02]	0.22
QI‐S4: Proportion of no prescription of antidepressants	0.96 [0.94–0.98]	**5.3 × 10** ^**−4**^	0.96 [0.94–0.99]	2.7 × 10^−3^
QI‐S5: Proportion of no prescription of anxiolytics or hypnotics	1.02 [1.01–1.04]	**8.6 × 10** ^**−4**^	1.03 [1.01–1.04]	**1.5 × 10** ^**−4**^
QI‐S6: Proportion of no prescription of mood stabilizers or antiepileptics	1.02 [1.01–1.04]	3.1 × 10^−3^	1.02 [1.00–1.04]	0.012
QI‐S7: Proportion of no prescription of anticholinergics	1.07 [1.06–1.09]	**4.7 × 10** ^**−18**^	1.08 [1.06–1.10]	**5.6 × 10** ^**−21**^
QI‐S8: Proportion of use of long‐acting injectable antipsychotics	1.08 [1.06–1.10]	**7.8 × 10** ^**−13**^	1.08 [1.06–1.10]	**1.4 × 10** ^**−13**^
QI‐S9: Proportion of clozapine treatment	1.09 [1.06–1.12]	**3.7 × 10** ^**−10**^	1.09 [1.06–1.12]	**1.7 × 10** ^**−10**^
QI‐S10: Proportion of modified electroconvulsive therapy	1.14 [1.11–1.17]	**1.5 × 10** ^**−25**^	1.11 [1.08–1.14]	**1.1 × 10** ^**−16**^
QI‐S11: Proportion of no prescription of psychotropic PRN medications	0.98 [0.97–1.00]	0.014	0.98 [0.96–0.99]	1.6 × 10^−3^

*Note:* Confounding factors, age, sex, and type of facilities, were adjusted. Significant differences are shown in bold (*p* < 1.4 × 10^−3^).

Abbreviations: CI, confidence interval; OR, odds ratio; PRN, pro re nata; QI, quality indicator; QI‐S, quality indicator of schizophrenia; TRS, treatment‐resistant schizophrenia.

#### 3.2.2 Major Depressive Disorder

Logistic regression analysis was conducted to assess the association between participation in the EGUIDE training (as the independent variable) and each QI for patients with depression, adjusting for age, sex, and facility type. The analysis revealed significant changes in three QIs: QI‐D1, QI‐D5 (cognitive behavioral therapy [CBT]), and QI‐D6 (mECT) (all *p* < 1.4 × 10^−3^) (Table 3).

**TABLE 3:** Longitudinal changes in QIs following institutional participation in the EGUIDE project for major depressive disorder.

QIs	Unadjusted OR [95% CI]	*p*	Adjusted OR [95% CI]	*p*
QI‐D1: Proportion of assessment of severity diagnosis	1.18 [1.16–1.21]	**5.1 × 10** ^**−43**^	1.20 [1.17–1.23]	**1.0 × 10** ^**−34**^
QI‐D2: Proportion of antidepressant monotherapy without other psychotropics	0.97 [0.94–1.00]	0.078	0.97 [0.94–1.01]	0.14
QI‐D3: Proportion of antidepressant monotherapy	1.01 [0.99–1.03]	0.42	1.01 [0.99–1.03]	0.28
QI‐D4: Proportion of no prescription of anxiolytics or hypnotics	1.00 [0.98–1.03]	0.71	1.01 [0.98–1.03]	0.61
QI‐D5: Proportion of cognitive‐behavioral therapy	0.82 [0.74–0.91]	**1.9 × 10** ^**−4**^	0.83 [0.74–0.92]	**4.2 × 10** ^**−4**^
QI‐D6: Proportion of modified electroconvulsive therapy	1.13 [1.10–1.16]	**1.2 × 10** ^**−21**^	1.10 [1.07–1.13]	**3.0 × 10** ^**−13**^
QI‐D7: Proportion of no prescription of psychotropic PRN medications	1.02 [1.00–1.04]	0.097	1.01 [0.99–1.04]	0.19

*Note:* Confounding factors, age, sex, and type of facilities, were adjusted. Significant differences are shown in bold (*p* < 1.4 × 10^−3^).

Abbreviations: CI, confidence interval; OR, odds ratio; PRN, pro re nata; QI, quality indicator; QI‐D, quality indicator of major depressive disorder.

**FIGURE 2:**
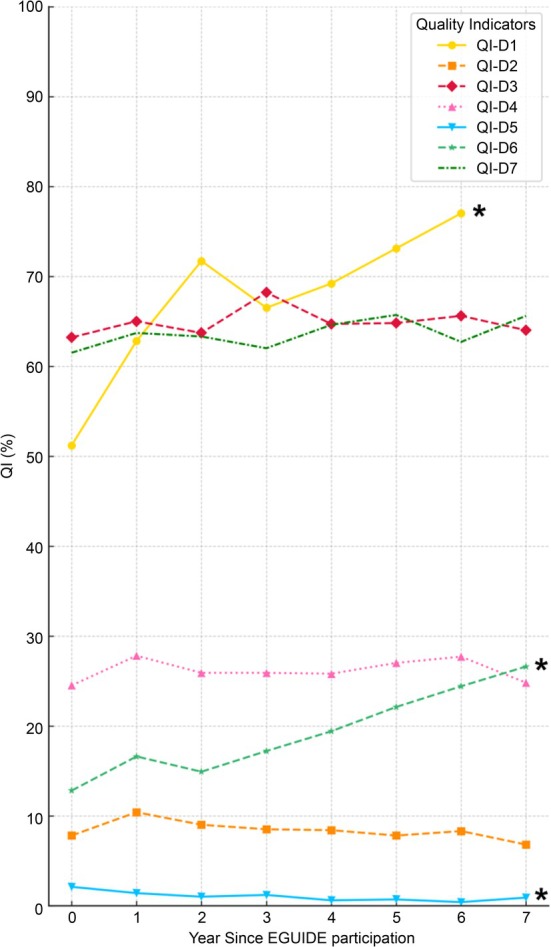
Trends in quality indicators for major depressive disorder treatment over the pre‐participation period and seven years post‐participation. This figure depicts the quality indicators (QIs) for patients with major depressive disorder enrolled in the EGUIDE Project. Data were collected from the period immediately preceding the intervention (Year 0) and for seven years following the intervention. The QIs included QI‐D1 (assessment of severity diagnosis), QI‐D2 (antidepressant monotherapy without other psychotropics), QI‐D3 (antidepressant monotherapy), QI‐D4 (no prescription of anxiolytics or hypnotics), QI‐D5 (cognitive‐behavioral therapy), QI‐D6 (modified electroconvulsive therapy), and QI‐D7 (no prescription of psychotropic PRN medications). Notably, data for QI‐D1 were collected starting from 2017, resulting in seven years of data collection for this specific indicator. The total number of patients was 9805, and the facility types were university hospitals (50.8%), public hospitals (22.4%), and private hospitals (26.7%). The QIs marked with an asterisk (*) indicate a statistically significant change over time, as determined by logistic regression analysis adjusted for age, sex, and facility type (*p* < 0.0014; Bonferroni‐corrected threshold: 0.05/36 comparisons).

In terms of percentage changes over time, QI‐D1 markedly increased from 51.2% to 77.0% (OR 1.20, 95% CI 1.17–1.23, *p* = 1.0 × 10^−34^), and QI‐D6 increased from 12.8% to 26.6% (OR 1.10, 95% CI 1.07–1.13, *p* = 3.0 × 10^−13^) (Figure 2, Table 3).

In contrast, QI‐D5, although statistically significant, showed a decreasing trend, with an OR of 0.83 (95% CI 0.74–0.92, *p* = 4.2 × 10^−4^), and the QI decreased from 2.1% to 0.9%.

## 4 Discussion

This study evaluated the long‐term impact of guideline training through the EGUIDE Project on the implementation of QIs for the treatment of schizophrenia and major depressive disorder. Multiple QIs significantly improved in both disorders, confirming the effectiveness of the guideline training.

A previous study [14] showed that physicians who participated in the EGUIDE training gradually improved in several QIs over a period of four years. In contrast to the previous study, the present study targeted entire facilities, including physicians who had not participated in the EGUIDE training, and evaluated the relationship between the number of years since participation in the EGUIDE training and QI performance using large‐scale data spanning up to eight years. In addition to the individual physician effects observed in the previous study, the proportion of improved QIs for schizophrenia substantially increased from 37.5% (three out of eight items) to 63.6% (7 out of 11 items). Notably, significant improvements were observed with the use of long‐acting injectable antipsychotics, treatment with Clozapine, and mECT, which had not shown improvement in the previous study (Table 2). For major depressive disorder, a significant improvement was also confirmed in the mECT item, which was not observed in the previous study (Table 3). These results strongly suggest that the presence of physicians who participated in the EGUIDE training led not only to enhancement of individual knowledge but also to a diffusion or spillover effect of knowledge and practices among healthcare professionals within the facility, including nonparticipating physicians and other staff, thereby improving the overall clinical culture of the facility [27]. This supports the importance of continuously implementing educational interventions to improve QIs and promote their dissemination.

Regarding QIs that showed common improvements across both disorders, QI‐S1 (assessment of TRS diagnosis) for schizophrenia and QI‐D1 (assessment of severity diagnosis) for major depressive disorder are both evaluation items conducted at the very initial stages of treatment, particularly during the first visit or hospitalization. These are crucial diagnostic processes directly linked to treatment decisions and can be performed independently by physicians, suggesting that changes in physicians' knowledge and awareness following guideline training are likely to translate directly into clinical behavior. Furthermore, QI‐S10 and QI‐D6, which relate to mECT, showed significant improvement in both disorders. Since mECT is a medical procedure that requires facility equipment and systems, this improvement suggests that EGUIDE training fosters not only changes in individual physician awareness but also indirectly influences the overall treatment culture of the facility. Furthermore, in the treatment for schizophrenia, significant improvements were observed in items related to the appropriate use of psychotropic drugs, such as antipsychotic monotherapy without the co‐prescription of other psychotropics (QI‐S2), the nonprescription of anxiolytics or hypnotics (QI‐S5), and the nonprescription of anticholinergics (QI‐S7). Regarding QI‐S2, reports from Western countries indicate that monotherapy rates reach 70%–85% [28]. However, these definitions primarily focus on avoiding the co‐prescription of multiple antipsychotics and do not necessarily exclude the co‐prescription of other psychotropics, such as anxiolytics or antidepressants. In contrast, the QI‐S2 in this study was evaluated based on a strict definition that excludes the co‐prescription of all such psychotropics. Although the monotherapy rate remained at 22.5% even seven years after the training, a steady improvement from before the training (15.7%) was observed. For QI‐S5, the long‐term use of benzodiazepines is also a concern in Western countries. For instance, the UK NICE guidelines generally do not recommend the co‐prescription of benzodiazepines for schizophrenia [29]. In this study, the rate of nonprescription of anxiolytics or hypnotics improved from 31.7% before the training to 37.7% after the training, suggesting progress toward internationally recommended levels. A domestic claims data study on patients with major depressive disorder reported a decrease in anxiolytic and hypnotic prescriptions from 83.7% to 79.8% between 2012 and 2018 [30]. It is possible that the observed decrease in anxiolytic and hypnotic prescriptions in this study was not solely attributable to the effects of the EGUIDE training. Regarding QI‐S7 (nonprescription of anticholinergics), the prescription rate also decreased annually from before the training, dropping to approximately 20% after seven years, which is comparable to rates reported in Western countries [31]. These results suggest that the EGUIDE Project contributed to the reduction of inappropriate polypharmacy.

However, not all the QIs showed any improvement. The nonprescription of PRN drugs for both schizophrenia (QI‐S11) and major depressive disorder (QI‐D7) showed no significant improvement. This suggests that PRN drugs remain clinically necessary for managing acute psychiatric symptoms in many cases, indicating a persistent discrepancy between the guideline recommendations and actual clinical practice. Furthermore, a significant decrease in the QI‐D5 (CBT) for major depressive disorder after the training is a critical issue. The extremely low implementation rate of CBT (approximately 1%) in Japan likely reflects challenges such as slow dissemination of CBT, a shortage of trained specialists, and difficulties in securing continuous session time [32]. Nonetheless, further efforts are needed to promote its implementation.

A limitation of this study was the lack of a control group, making it impossible to definitively rule out the possibility that the observed QI improvements merely reflected natural changes in treatment practices over time, irrespective of the influence of EGUIDE training. Because informed consent was not obtained from nonparticipating psychiatrists, their exact numbers and patient information were not collected. However, patient assignment at medical facilities is not systematically allocated based on whether physicians have participated in the training or not. Therefore, the number of patients handled per physician is expected to be similar across participating and nonparticipating physicians. Given that 1421 physicians participated in the training and treated 17 498 patients, while nonparticipating physicians treated 11 930 patients, we estimate that approximately 969 nonparticipating psychiatrists were involved in patient care during the study period. However, the observation of improvements in a greater number of QIs than reported in the previous study [14], along with the improvement in QIs related to facility‐wide systems, such as mECT, suggests the existence of a facility‐level impact of EGUIDE training—namely, a spillover effect—that cannot be fully explained by natural changes or the cumulative effect of individual participating physicians alone [27]. Furthermore, this study evaluated annual changes in QIs; however, clinical outcomes such as patient satisfaction, functional recovery, and frequency of adverse effects were not included. Future research should integrate broader outcome measures to comprehensively evaluate the impact of guideline training on patient care.

## 5 Conclusion

This multi‐institutional study observed long‐term, facility‐wide improvements in guideline‐recommended treatments for both schizophrenia and major depressive disorder after participation in the EGUIDE training. These findings suggest a diffusion or spillover effect, in which knowledge spreads across the clinical team, including nonparticipating physicians, thereby enhancing the quality of institutional care. Despite the lack of a control group, these findings highlight the significant potential of the EGUIDE to improve psychiatric healthcare at the facility level.

## Author Contributions

N.H. and H.M. were involved in data collection and analysis and wrote the first draft of the manuscript. Y.A., T.H., K.Y., N.H., S.O., T.T., Y.K., T.N., Y.Y., J.M., S.I., T.O., K.O., S.N., H.H., and N.Y.‐F. contributed to the interpretation of the data and data collection. K.W. and K.I. were involved in the study design and contributed to the interpretation of the data. R.H. supervised the entire project, collected the data, and was involved in the data's design, analysis, and interpretation. All authors contributed to and approved the final article.

## Ethics Statement

The study was conducted with the approval of the National Center of Neurology and Psychiatry Ethics Committee. The study was conducted in accordance with the Declaration of Helsinki of the World Medical Association.

## Consent

Written informed consent was obtained from each psychiatrist.

## Conflicts of Interest

The authors declare no conflicts of interest.

## Supporting information


**Table S1:** npr270067‐sup‐0001‐TableS1.docx.

**Table S2:** npr270067‐sup‐0002‐TableS2.docx.

**Table S3:** npr270067‐sup‐0003‐TableS3.docx.

**Table S4:** npr270067‐sup‐0004‐TableS4.docx.

## Data Availability

The data are not publicly available due to privacy and ethical restrictions (i.e., we did not obtain informed consent on the public availability of raw data).
