# Supplementary material for: Long‐Term Impact of EGUIDE Training on Facility‐Wide Guideline Adherence Rate in Schizophrenia and Major Depressive Disorder: A Multicenter Study
Source: Neuropsychopharmacol Rep. 2025 Oct 28;45(4):e70067. doi: 10.1002/npr2.70067 (PMC12560011; doi:10.1002/npr2.70067)
Supplement: Supplementary file 1 — Table S1: npr270067‐sup‐0001‐TableS1.docx. [file NPR2-45-e70067-s002.docx]

QIs

Description

QI-S1

Proportion of Assessment of TRS Diagnosis

QI-S2

Proportion of Antipsychotic monotherapy without other psychotropics

QI-S3

Proportion of Antipsychotic monotherapy

QI-S4

Proportion of No prescription of Antidepressant

QI-S5

Proportion of No prescription of anxiolytics or hypnotics

QI-S6

Proportion of No prescription of mood stabilizers or antiepileptics

QI-S7

Proportion of No prescription of anticholinergics

QI-S8

Proportion of Use of Long-acting injectable antipsychotics

QI-S9

Proportion of Clozapine treatment

QI-S10

Proportion of Modified electroconvulsive therapy

QI-S11

Proportion of No prescription of psychotropic pro re nata medications

Abbreviatons: QI, Quality Indicator; S, Schizophrenia; TRS, Treatment Resistant

Schizophrenia

Supplementary Table1. Quality Indicators (QIs) for Schizophrenia Treatment in

the EGUIDE project
