# Supplementary material for: Long‐Term Impact of EGUIDE Training on Facility‐Wide Guideline Adherence Rate in Schizophrenia and Major Depressive Disorder: A Multicenter Study
Source: Neuropsychopharmacol Rep. 2025 Oct 28;45(4):e70067. doi: 10.1002/npr2.70067 (PMC12560011; doi:10.1002/npr2.70067)
Supplement: Supplementary file 2 — Table S2: npr270067‐sup‐0002‐TableS2.docx. [file NPR2-45-e70067-s004.docx]

QIs

Description

QI-D1

Proportion of Assessment of severity diagnosis

QI-D2

Proportion of Antidepressant monotherapy without other psychotropics

QI-D3

Proportion of Antidepressant monotherapy

QI-D4

Proportion of No prescription of anxiolytics or hypnotics

QI-D5

Proportion of Cognitive-behavioural therapy

QI-D6

Proportion of Modified electroconvulsive therapy

QI-D7

Proportion of No prescription of psychotropic pro re nata medications

Supplementary Table2. Quality Indicators (QIs) for Major depressive disorder

Treatment in the EGUIDE project

Abbreviatons: QI, Quality Indicator
