# Supplementary material for: Long‐Term Impact of EGUIDE Training on Facility‐Wide Guideline Adherence Rate in Schizophrenia and Major Depressive Disorder: A Multicenter Study
Source: Neuropsychopharmacol Rep. 2025 Oct 28;45(4):e70067. doi: 10.1002/npr2.70067 (PMC12560011; doi:10.1002/npr2.70067)
Supplement: Supplementary file 3 — Table S3: npr270067‐sup‐0003‐TableS3.docx. [file NPR2-45-e70067-s001.docx]

Supplementary Table 3. Number of Patients with Schizophrenia by Year of EGUIDE Participation and Follow-up Period

Note: Year 0 represents pre-participation data; Years 1–7 indicate post-participation follow-ups. The data reflected the discharge records of 19,623 patients with major depressive disorder from 298 facilities. NA indicates data not available due to the 2023 data collection endpoint (e.g., a 7-year follow-up for the 2017 enrollment was unavailable).

Abbreviations: NA, not available; EGUIDE, Effectiveness Research on the Dissemination and Education of Psychiatric Clinical Practice Guidelines.
