# Supplementary material for: Long‐Term Impact of EGUIDE Training on Facility‐Wide Guideline Adherence Rate in Schizophrenia and Major Depressive Disorder: A Multicenter Study
Source: Neuropsychopharmacol Rep. 2025 Oct 28;45(4):e70067. doi: 10.1002/npr2.70067 (PMC12560011; doi:10.1002/npr2.70067)
Supplement: Supplementary file 4 — Table S4: npr270067‐sup‐0004‐TableS4.docx. [file NPR2-45-e70067-s003.docx]

Supplementary Table 4. Number of Patients with MDD by Year of EGUIDE Participation and Follow-up Period

Note: Year 0 represents pre-participation data; Years 1–7 indicate post-participation follow-ups. The data reflected the discharge records of 9,805 patients with major depressive disorder from 298 facilities. NA indicates data not available due to the 2023 data collection endpoint (e.g., a 7-year follow-up for the 2017 enrollment was unavailable).

Abbreviations: MDD, Major depressive disorder; NA, not available; EGUIDE, Effectiveness Research on the Dissemination and Education of Psychiatric Clinical Practice Guidelines.
